# Increased Circulating Adiponectin in Response to Thiazolidinediones: Investigating the Role of Bone Marrow Adipose Tissue

**DOI:** 10.3389/fendo.2016.00128

**Published:** 2016-09-21

**Authors:** Richard J. Sulston, Brian S. Learman, Bofeng Zhang, Erica L. Scheller, Sebastian D. Parlee, Becky R. Simon, Hiroyuki Mori, Adam J. Bree, Robert J. Wallace, Venkatesh Krishnan, Ormond A. MacDougald, William P. Cawthorn

**Affiliations:** ^1^University/British Heart Foundation Centre for Cardiovascular Science, The Queen’s Medical Research Institute, University of Edinburgh, Edinburgh, UK; ^2^Department of Molecular & Integrative Physiology, University of Michigan Medical School, Ann Arbor, MI, USA; ^3^Program in Cellular and Molecular Biology, University of Michigan Medical School, Ann Arbor, MI, USA; ^4^Department of Orthopaedics, University of Edinburgh, Edinburgh, UK; ^5^Musculoskeletal Research, Lilly Research Laboratories, Indianapolis, IN, USA; ^6^Department of Internal Medicine, University of Michigan Medical School, Ann Arbor, MI, USA

**Keywords:** bone marrow adipose tissue, white adipose tissue, brown adipose tissue, thiazolidinedione, rosiglitazone, adiponectin, beige adipocyte, UCP1

## Abstract

**Background:**

Bone marrow adipose tissue (MAT) contributes to increased circulating adiponectin, an insulin-sensitizing hormone, during caloric restriction (CR), but whether this occurs in other contexts remains unknown. The antidiabetic thiazolidinediones (TZDs) also promote MAT expansion and hyperadiponectinemia, even without increasing adiponectin expression in white adipose tissue (WAT).

**Objectives:**

To test the hypothesis that MAT expansion contributes to TZD-associated hyperadiponectinemia, we investigated the effects of rosiglitazone, a prototypical TZD, in wild-type (WT) or *Ocn*-Wnt10b mice. The latter resist MAT expansion during CR, leading us to postulate that they would also resist this effect of rosiglitazone.

**Design:**

Male and female WT or *Ocn*-Wnt10b mice (C57BL/6J) were treated with or without rosiglitazone for 2, 4, or 8 weeks, up to 30 weeks of age. MAT content was assessed by osmium tetroxide staining and adipocyte marker expression. Circulating adiponectin was determined by ELISA.

**Results:**

In WT mice, rosiglitazone caused hyperadiponectinemia and MAT expansion. Compared to WT mice, *Ocn*-Wnt10b mice had significantly less MAT in distal tibiae and sometimes in proximal tibiae; however, interpretation was complicated by the leakage of osmium tetroxide from ruptures in some tibiae, highlighting an important technical consideration for osmium-based MAT analysis. Despite decreased MAT in *Ocn-*Wnt10b mice, circulating adiponectin was generally similar between WT and *Ocn*-Wnt10b mice; however, in females receiving rosiglitazone for 4 weeks, hyperadiponectinemia was significantly blunted in *Ocn-*Wnt10b compared to WT mice. Notably, this was also the only group in which tibial adiponectin expression was lower than in WT mice, suggesting a close association between MAT adiponectin production and circulating adiponectin. However, rosiglitazone significantly increased adiponectin protein expression in WAT, suggesting that WAT contributes to hyperadiponectinemia in this context. Finally, rosiglitazone upregulated uncoupling protein 1 in brown adipose tissue (BAT), but this protein was undetectable in tibiae, suggesting that MAT is unlikely to share thermogenic properties of BAT.

**Conclusion:**

TZD-induced hyperadiponectinemia is closely associated with increased adiponectin production in MAT but is not prevented by the partial loss of MAT that occurs in *Ocn-*Wnt10b mice. Thus, more robust loss-of-MAT models are required for future studies to better establish MAT’s elusive functions, both on an endocrine level and beyond.

## Introduction

Adipose tissue is typically classified into two broad subtypes, white adipose tissue (WAT) and brown adipose tissue (BAT). WAT is commonly known for its role in energy storage and release and is now established as a major endocrine organ. BAT also mediates some endocrine functions but is more widely known for its ability to mediate adaptive thermogenesis (1). Through these functions, both WAT and BAT impact metabolic homeostasis; hence, the global burden of obesity and metabolic disease has motivated extensive study of these tissues (2).

In addition to WAT and BAT, adipocytes also exist in the bone marrow, and such marrow adipose tissue (MAT) has been estimated to account for over 10% of total adipose tissue mass in lean, healthy humans (3). We recently revealed that MAT characteristics are region-specific, such that MAT can be classified into two broad subtypes: regulated MAT (rMAT), which exists in more proximal skeletal sites and consists of adipocytes interspersed with hematopoietic BM; and constitutive MAT (cMAT), which exists in more distal regions (e.g., distal tibia, caudal vertebrae) and appears histologically similar to WAT, with few visible hematopoietic cells (4). These MAT subtypes also differ in their lipid composition and response to external stimuli (4). Both rMAT and cMAT appear to be developmentally and functionally distinct to WAT and BAT, and therefore MAT may represent a third general class of adipose tissue (5, 6). Despite these advances, knowledge of MAT formation and function remains relatively limited (2). However, research from others and us suggests that, like WAT, MAT is an endocrine organ that can exert local and systemic effects (3, 7).

The appreciation of WAT as an endocrine organ derives largely from the discovery in the mid-1990s of two adipocyte-derived hormones, leptin and adiponectin, each of which impacts metabolic homeostasis (8–10). These two hormones have since been mentioned in over 40,000 publications, reflecting the extensive depth of WAT research. Circulating leptin concentrations correlate directly with body fat percentage and are therefore increased in obesity. In contrast, adiponectin concentrations are decreased in obesity and insulin-resistance; hence, hypoadiponectinemia is now an established biomarker for increased risk of cardiometabolic disease. Conversely, circulating adiponectin is elevated in conditions of leanness and insulin sensitivity, such as during caloric restriction (CR) (2). This counterintuitive observation has been dubbed the “adiponectin paradox”: why should circulating adiponectin increase when the amount of WAT, the presumed source of adiponectin, is decreased? Moreover, CR can cause hyperadiponectinemia without increasing expression or secretion of adiponectin from WAT (2), suggesting that other tissues contribute to this effect. Our recent research has highlighted a potential explanation for this paradox, revealing, unexpectedly, that MAT is a source of circulating adiponectin during CR (3).

Our studies into MAT and CR were motivated by the striking observation that, in contrast to WAT and BAT, MAT accumulates during CR (3, 11, 12). The biochemical phenotype of this CR-responsive MAT remains to be elucidated; however, we have since shown that these increases occur predominantly in regions of rMAT rather than cMAT (2). This phenomenon led us to investigate if MAT contributes to increased circulating adiponectin during CR. After confirming that MAT expresses and secretes adiponectin (3), we then tested if MAT accumulation is required for CR-associated hyperadiponectinemia. To do so, we used *Ocn*-Wnt10b mice, a transgenic model in which the secreted ligand, Wnt10b, is expressed in osteoblasts from the *Ocn* promoter (13). Because Wnt10b simulates osteoblastogenesis and inhibits adipogenesis, these mice have increased bone formation and decreased bone marrow volume (3, 13). We found that *Ocn-*Wnt10b mice also resist CR-associated MAT expansion, both in rMAT and cMAT (2, 3). Notably, hyperadiponectinemia during CR is also blunted in these mice, despite no differences in adiponectin expression in WAT (3). Finally, impaired MAT expansion also coincides with blunted hyperadiponectinemia in a separate mouse model of CR (14) and during CR in rabbits (12). Together, these observations suggest that MAT expansion is required for CR-induced hyperadiponectinemia, supporting the conclusion that MAT is a source of circulating adiponectin in this context.

To further investigate this endocrine function, we sought to determine if MAT also influences circulating adiponectin beyond CR. For example, increases in both MAT and circulating adiponectin occur in many other conditions, including aging, estrogen deficiency, type 1 diabetes, cancer treatment, and in response to fibroblast growth factor-21 or glucocorticoid therapy (2). Notably, these increases also coincide during treatment with thiazolidinediones (TZDs), a class of insulin-sensitizing, antidiabetic drugs that act as agonists for the nuclear hormone receptor, peroxisome proliferator-activated receptor gamma (PPARγ) (15, 16). Binding and subsequent activation of PPARγ causes it to activate the expression of its transcriptional targets, including adiponectin and other lipid-metabolism-associated genes such as fatty acid-binding protein 4 (*Fabp4*) (17). Thus, TZDs, such as rosiglitazone, significantly increase circulating adiponectin in rodent models and human patients (18, 19). Importantly, preclinical studies suggest that such hyperadiponectinemia is required for the full insulin-sensitizing effects of TZDs (20). Despite the beneficial effects of TZDs in improving glucose tolerance, their clinical use has been restricted owing to increased risk of myocardial infarction and bone fractures (21, 22); the latter may relate to the ability of TZDs to drive MAT expansion. If so, MAT expansion may be detrimental to the clinical utility of these drugs. However, it is notable that TZDs can increase circulating adiponectin without increasing adiponectin expression in WAT (23, 24), and that some studies suggest that TZD action is independent of WAT (25). Thus, given the contribution of MAT to increased circulating adiponectin during CR, we hypothesized that MAT also contributes to TZD-induced hyperadiponectinemia. If so, MAT expansion may play a role in the beneficial insulin-sensitizing effects of TZDs.

Herein, we investigated this hypothesis by feeding wild-type (WT) and *Ocn*-Wnt10b mice a Western diet to induce obesity and glucose intolerance, followed by treatment with rosiglitazone, a prototypical TZD. We postulated that, as in CR, these mice would resist TZD-induced MAT expansion. As secondary analyses, we also investigated the contribution of WAT to TZD-mediated hyperadiponectinemia and the possibility, suggested previously (26), that MAT has BAT-like properties. Our results shed light on MAT’s characteristics and highlight technical considerations that will be important for future research into MAT formation and function.

## Materials and Methods

### Animals and Animal Care

*Ocn*-Wnt10b mice (Wnt10b) or non-transgenic controls (WT) were on a C57BL/6J background and were bred in-house, as described previously (13). The University of Michigan Committee on the Use and Care of Animals approved all animal experiments, with daily care of mice and rabbits overseen by the Unit for Laboratory Animal Medicine (ULAM).

### Diets and Rosiglitazone (TZD) Treatment

Male WT (*n* = 18), male Wnt10b (*n* = 22), female WT (*n* = 20), and female Wnt10b (*n* = 15) mice were fed a standard laboratory chow diet (Research Diets, D12450B) from weaning until 10 weeks of age. From 10 to 22 weeks of age, all mice were fed a high-fat, high-sucrose Western diet (Research Diets D12079B), with the goal of promoting diet-induced obesity and glucose intolerance. The rationale for this was that, if *Ocn*-Wnt10b mice resisted hyperadiponectinemia, they might also be less responsive to the metabolic effects of TZD treatment. Thus, mice were fed a Western diet prior to TZD treatment, with the aim of allowing detection of metabolic improvements upon TZD administration. At 18 weeks of age, mice were fasted overnight; body mass and fasting glucose were then recorded and body fat, lean mass, and free fluid were measured in conscious mice using an NMR analyzer (Minispec LF90II; Bruker Optics, Billerica, MA, USA). These measurements were then used as a basis to assign mice to four evenly matched groups (with similar body mass, fat mass, and fasting glucose), with each group corresponding to a different duration of rosiglitazone (TZD) treatment. Mice were then treated with or without TZD from 22 to 30 weeks of age. TZD was administered in the diet (Research Diets D12112601) by supplementing diet D12450B with rosiglitazone at 0.175 mg/g diet; based on daily consumption of D12450B, this concentration was estimated to give a final dose per mouse of 15 mg/kg body mass per day. This approach is similar to that used in previous studies investigating rosiglitazone-induced MAT accumulation (26–28). The four experimental groups were as follows: control mice (0 weeks’ TZD), which continued to receive Western diet D12079B from 22 to 30 weeks of age; 2 weeks’ TZD mice, which received D12079B from 22 to 28 weeks and D12112601 from 28 to 30 weeks; 4 weeks’ TZD mice, which received D12079B from 22 to 26 weeks and D12112601 from 26 to 30 weeks; and 8 weeks’ TZD mice, which received D12112601 from 22 to 30 weeks. Numbers of mice per group and diet durations are described further in Table 1. At 29.5 weeks of age, blood glucose concentrations were recorded after an overnight fast to assess the effects of TZD treatment. At 30 weeks of age, serum was sampled, mice were humanely euthanized, and tissues were isolated for further analysis.

**Table 1:** **Summary of groups for control or TZD treatment**.

Weeks of TZD	Ages (weeks) fed Western diet (D12450B)	Ages (weeks) fed TZD diet (D12112601)	Group sizes for each sex and genotype
0	22–30	N/A	WT male, 5; *Ocn-*Wnt10b male, 5
			WT female, 6; *Ocn-*Wnt10b female, 4
2	22–28	28–30	WT male, 3; *Ocn-*Wnt10b male, 7
			WT female, 5; *Ocn-*Wnt10b female, 3
4	22–26	26–30	WT male, 5; *Ocn-*Wnt10b male, 5
			WT female, 5; *Ocn-*Wnt10b female, 3
8	N/A	22–30	WT male, 5; *Ocn-*Wnt10b male, 5
			WT female, 4; *Ocn-*Wnt10b female, 5

### Blood Collection and Serum Adiponectin Analysis

Blood was sampled from the lateral tail vein of mice using Microvette CB 300 capillary collection tubes (Sarstedt, Newton, NC, USA). Blood glucose was measured using an Accu-Chek Aviva glucometer. To obtain serum, blood samples were allowed to clot on ice for 2 h before centrifuging at 3,800 RCF for 5 min at 4°C. Serum adiponectin was determined using an ELISA kit (catalog no. MRP300) from R&D Systems (Bio-Techne Ltd., Abingdon, UK) according to the manufacturer’s instructions.

### Osmium Tetroxide Staining and μCT Analysis

Tibiae were isolated and, after removal of external soft tissue, fixed in formalin at 4°C. Fixed tibiae were decalcified in 14% EDTA for 14 days and then washed in Sorensen’s Phosphate buffer (81 mM KH_2_PO_4_, 19 mM Na_2_HPO_4_ ⋅ 7H_2_O, pH 7.4). Decalcified tibiae were stored in Sorensen’s Phosphate buffer at 4°C until ready to be stained with osmium tetroxide. To do so, osmium tetroxide solution (2% w/v; Agar Scientific, UK) was diluted 1:1 in Sorensen’s Phosphate buffer. Tibiae were then stained in this 1% osmium tetroxide solution for 48 h at room temperature, then washed, and stored in Sorensen’s Phosphate buffer at 4°C prior to micro computed tomography (μCT) analysis.

### Micro Computed Tomography Analysis

Layers of four to five stained tibiae were arranged in parallel in 1% agarose in a 30-mL universal tube and mounted in a Skyscan 1172 desktop micro CT (Bruker, Kontich, Belgium). The samples were then scanned through 360° using a step of 0.40° between exposures. A voxel resolution of 12.05 μm was obtained in the scans using the following control settings: 54 kV source voltage, 185 μA source current with an exposure time of 885 ms. A 0.5-mm aluminum filter and two-frame averaging were used to optimize the scan. After scanning, the data were reconstructed using Skyscan software NRecon v1.6.9.4 (Bruker, Kontich, Belgium). The reconstruction thresholding window was optimized to encapsulate the target image. Volumetric analysis was performed using CT Analyser v1.13.5.1 (Bruker, Kontich, Belgium).

### Real-time Quantitative PCR

RNA was extracted from tissue using RNA STAT60 reagent (Tel-Test, Inc.) according to the manufacturer’s instructions. Synthesis of cDNA was done using TaqMan reverse transcription reagents (Thermo Fisher Scientific) using 1 μg of RNA template per reaction, as per manufacturer’s instructions. Transcript expression was then analyzed by quantitative PCR (qPCR) in 10 μL duplicate reactions using qPCRBIO SyGreen Mix (part number PB20.11; PCR Biosystems, UK) and 1–4 μL of cDNA template. Reactions were loaded into 384-well qPCR plates (part number 72.1985.202; Sarstedt, UK) and run on a Light Cycler 480 (Roche). Transcript expression was calculated based on a cDNA titration loaded on each plate and was presented relative to expression of the housekeeping gene *Ppia*. Primers for *Adipoq* and *Ppia* were described and validated previously (3).

### Immunoblot Analysis

Frozen tissue was processed as described previously (12). The resulting protein lysates were separated by size using gradient (4–12%) polyacrylamide gels (BioRad). Protein was then transferred to Immobilon-FL membrane (Millipore) for 150 min at 350 mA, 4°C, using a Criterion wet-transfer system (BioRad). Post-transfer, the membranes were blocked in 5% milk for 1 h at room temperature, then immunoblotted with primary antibody in 5% bovine serum albumin overnight at 4°C. Membranes were then incubated in 1:15,000-diluted fluorescently labeled secondary antibody (LiCor) for 1 h at room temperature. Signal was detected using the LiCor Odyssey system and band intensities quantified using LiCor Image Studio Lite software. The following primary antibodies were used: rabbit anti-adiponectin antibody (Sigma, A6354-200UL) diluted 1:1,000 in 5% BSA; rabbit anti-uncoupling protein 1 (UCP1) antibody (Sigma, U6382) diluted 1:10,000 in 2.5% milk; and rabbit anti-β-actin antibody (Abcam, ab8227) diluted 1:1,000 in 5% BSA.

### Data Presentation and Statistical Analysis

Data are presented as box and whisker plots overlaid with individual data points for each animal. Boxes indicate the 25th and 75th percentiles; whiskers display the range; and horizontal lines in each box represent the median. Group sizes are described in Table 1. Statistical analysis was done using GraphPad Prism 6 software, with significant differences assessed by two-way ANOVA using a Tukey or Sidak *post hoc* test for multiple comparisons, as appropriate. A *P*-value <0.05 was considered statistically significant. A significant influence of TZD treatment, across all treatment groups, is indicated by †. For multiple comparisons, asterisks (*) indicate significant differences between genotypes within each TZD group, while hash signs (#) indicate significance between TZD-treated and non-TZD-treated controls within each genotype.

## Results

### Effects of TZD on WAT Mass and Fasting Glucose Do Not Differ between WT and *Ocn-*Wnt10b Mice

**Figure 1:**
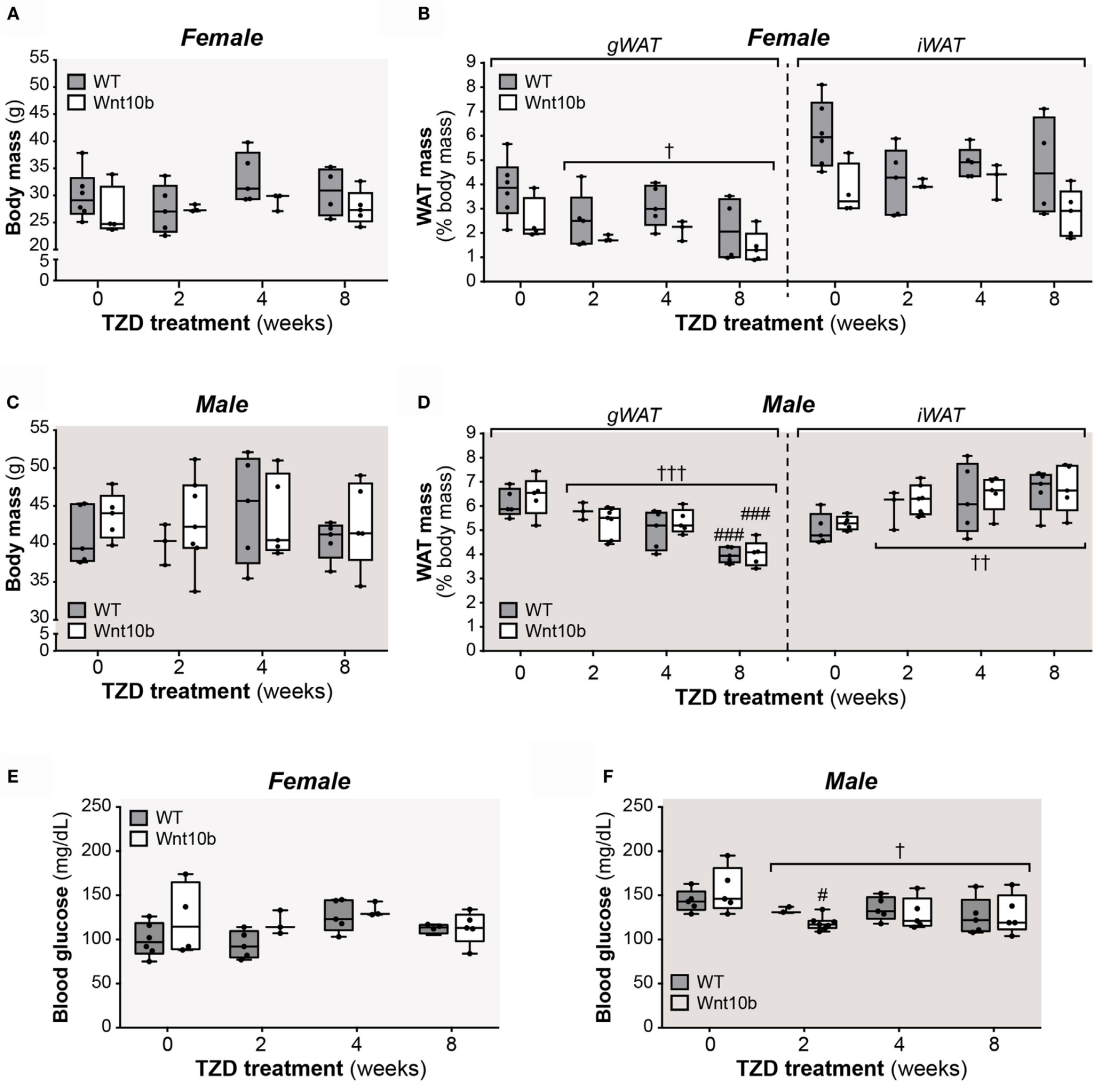
**Effects of rosiglitazone on body mass, fat mass, and fasting glucose**. Male and female mice were fed a control Western diet only or a Western diet supplemented with rosiglitazone for 2, 4, or 8 weeks, as described in Section “Materials and Methods.” **(A,C)** Body masses of females **(A)** and males **(C)** were recorded prior to necropsy at 30 weeks of age. **(B,D)** Masses of iWAT and gWAT were recorded at necropsy for female **(B)** and male mice **(D)** and are shown as percentage of total body mass. **(E,F)** Fasting blood glucose, as recorded at 29.5 weeks of age. For **(A–F)**, a significant influence of TZD treatment, across all treatment groups, is indicated by † (*P* < 0.05), †† (*P* < 0.01) or ††† (*P* < 0.001). Within each genotype, significant differences between untreated controls (0-week TZD) and individual durations of TZD treatment are indicated by ### (*P* < 0.001). Within each TZD treatment group (0-, 2-, 4-, or 8-week TZD), there were no statistically significant differences between WT and *Ocn*-Wnt10b mice.

Circulating adiponectin is decreased in states of obesity and glucose intolerance (29), while TZDs modulate both fat mass and glucose homeostasis. Therefore, after treating WT and *Ocn-*Wnt10b mice with or without rosiglitazone for 2, 4, or 8 weeks, we first assessed body mass, fat mass, and fasting glucose, primarily as readouts of TZD action but also as parameters that might influence circulating adiponectin. While body masses of female or male mice did not differ with TZD treatment (Figures 1A,C), across both genotypes, TZD was associated with significant loss of gonadal WAT (gWAT), a visceral adipose depot, in both females (*P* = 0.027) and males (*P* = 0.006) (Figures 1B,D). Conversely, the mass of inguinal WAT (iWAT), a subcutaneous depot, significantly increased with TZD treatment in males (*P* = 0.002) (Figure 1D). Analysis of fasting glucose revealed no effects of TZD in female mice (Figure 1E), whereas TZD significantly decreased fasting glucose across WT and *Ocn-*Wnt10b males (Figure 1F). These findings are consistent with previous studies demonstrating that rosiglitazone decreases gWAT mass (26, 27), increases subcutaneous WAT (30), and ameliorates hyperglycemia (16); however, it is unclear why rosiglitazone influenced iWAT mass and fasting glucose in males but not in females. In contrast to these effects of rosiglitazone, body mass, WAT mass, or fasting glucose did not differ between WT and *Ocn*-Wnt10b mice within each TZD treatment group (Figures 1A–F). Thus, differences in peripheral adiposity or glucose homeostasis, which can influence circulating adiponectin, did not occur between WT and *Ocn*-Wnt10b mice.

### TZD Increases rMAT and cMAT Volume in WT Mice, and These Effects Are Partially Blunted in *Ocn-*Wnt10b Mice

**Figure 2:**
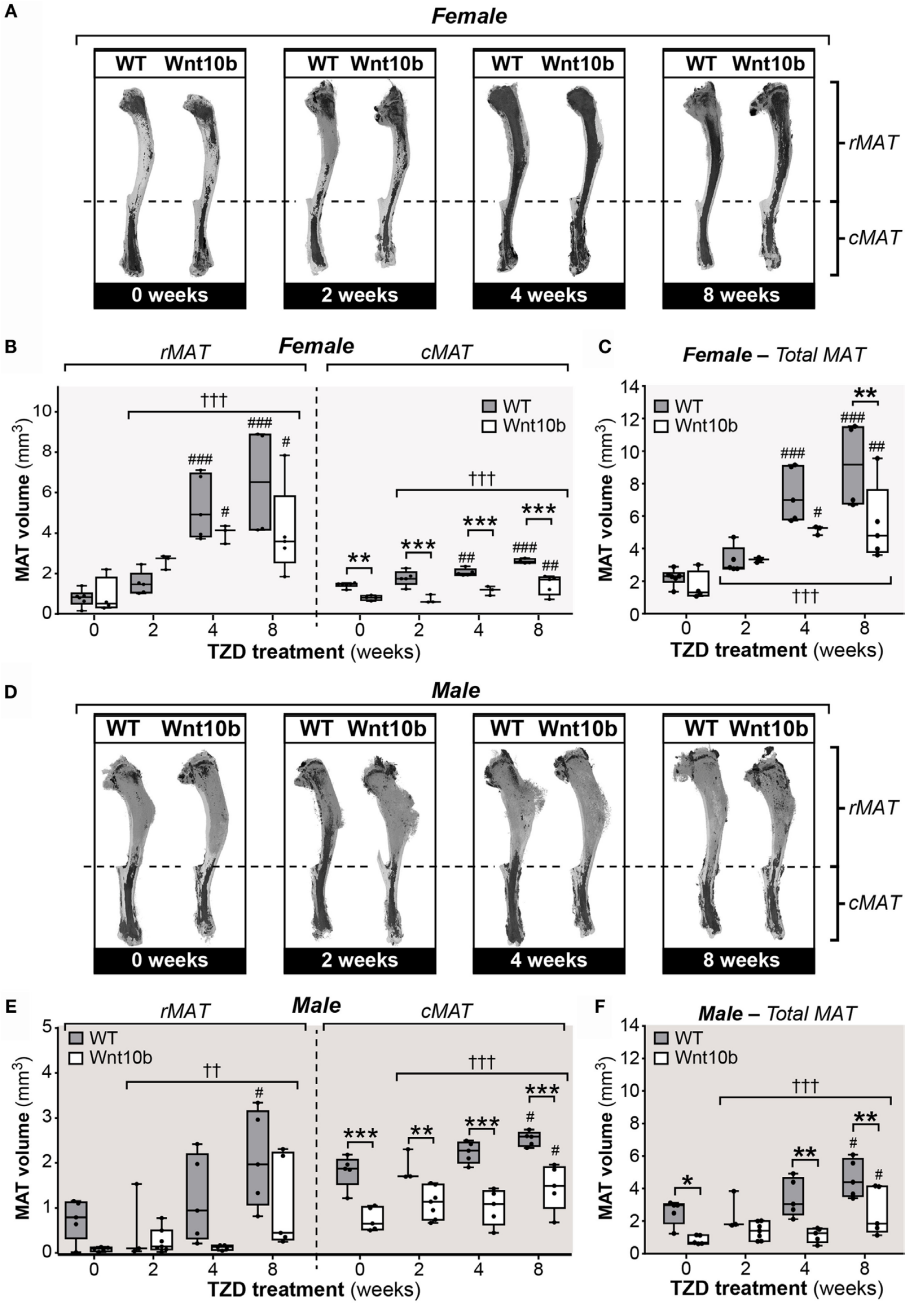
**Rosiglitazone increases rMAT and cMAT volume in WT mice and cMAT expansion is blunted in *Ocn*-Wnt10b mice**. Male and female tibiae were dissected at necropsy. For each mouse, one tibia was decalcified in EDTA, stained with 1% osmium tetroxide, and analyzed by μCT scanning, as described in Section “Materials and Methods.” **(A,D)** Representative μCT scans of stained tibiae from female **(A)** and male mice **(D)**. The dashed line indicates the tibia–fibula junction as the boundary between rMAT and cMAT. **(B,C,E,F)** For female **(B,C)** and male mice **(E,F)**, μCT scans were used to determine the volumes of rMAT (proximal to tibia–fibula junction), cMAT (distal to tibia–fibula junction), and total MAT (whole tibia), as indicated. In **(B,E)**, volumes of rMAT and cMAT are presented on the same *y*-axis scale. A significant influence of TZD treatment, across all treatment groups, is indicated by † (*P* < 0.05), †† (*P* < 0.01) or ††† (*P* < 0.001). Within each genotype, significant differences between TZD-treated mice and untreated controls are indicated by # (*P* < 0.05), ## (*P* < 0.01) or ### (*P* < 0.001). Within each TZD treatment group, statistically significant differences between WT and *Ocn-*Wnt10b mice are indicated by * (*P* < 0.05), ** (*P* < 0.01), or *** (*P* < 0.001).

Having characterized these peripheral metabolic parameters, we next investigated the impact of TZD on MAT expansion and whether this differs between WT and *Ocn-*Wnt10b mice. To do so, we first used an approach based on staining tibiae with osmium tetroxide, which covalently binds to unsaturated lipids within bone marrow and thereby acts as a strong contrast agent for MAT detection by μCT (31). As shown in Figure 2A, osmium staining in the proximal diaphysis, corresponding to rMAT, was markedly increased in females of both genotypes following 4- or 8-week TZD treatment. Statistical analyses confirmed that, across all groups of females, TZD significantly influenced the volume of rMAT, as well as that of cMAT and total MAT (*P* < 0.0001 for each) (Figures 2B,C). Multiple comparisons were then made to assess the effect of each duration of TZD treatment in each genotype of mice, relative to untreated controls. This revealed that, in female WT mice, TZD treatment for 4 or 8 weeks significantly increased rMAT volume in proximal tibiae and cMAT volume in distal tibiae (Figures 2A,B). Thus, total tibial MAT volume was also significantly increased by 4- or 8-week TZD in WT females (Figure 2C). Similar effects of TZD were also observed in *Ocn*-Wnt10b females (Figures 2A–C), and rMAT volume did not significantly differ between genotypes (Figure 2B). However, cMAT volume was markedly lower in *Ocn-*Wnt10b females within each TZD treatment group (Figure 2B). Total tibial MAT volume was also significantly decreased in *Ocn*-Wnt10b compared to WT females following 8 weeks’ TZD (Figure 2C).

Compared to the female mice, osmium staining was less pronounced in the rMAT of TZD-treated males (Figure 2D). Nevertheless, statistical analysis across all groups of males revealed a significant influence of TZD on the volumes of rMAT (*P* = 0.0015), cMAT (*P* = 0.0006), and total MAT (*P* = 0.0003) (Figures 2E,F). Multiple comparisons further revealed that, in WT males, treatment with TZD for 8 weeks, but not shorter durations, significantly increased volumes of rMAT, cMAT, and total MAT (Figures 2E,F). This also occurred for cMAT and total MAT, but not rMAT, in *Ocn-*Wnt10b males (Figures 2E,F). As found for female mice, male *Ocn-*Wnt10b mice had significantly decreased cMAT volume compared to their WT counterparts (Figure 2E), and this also occurred for total MAT volume in the 0-, 4-, and 8-week TZD groups (Figure 2F).

### Cortical Bone Ruptures Significantly Decrease Detectable rMAT Volume

**Figure 3:**
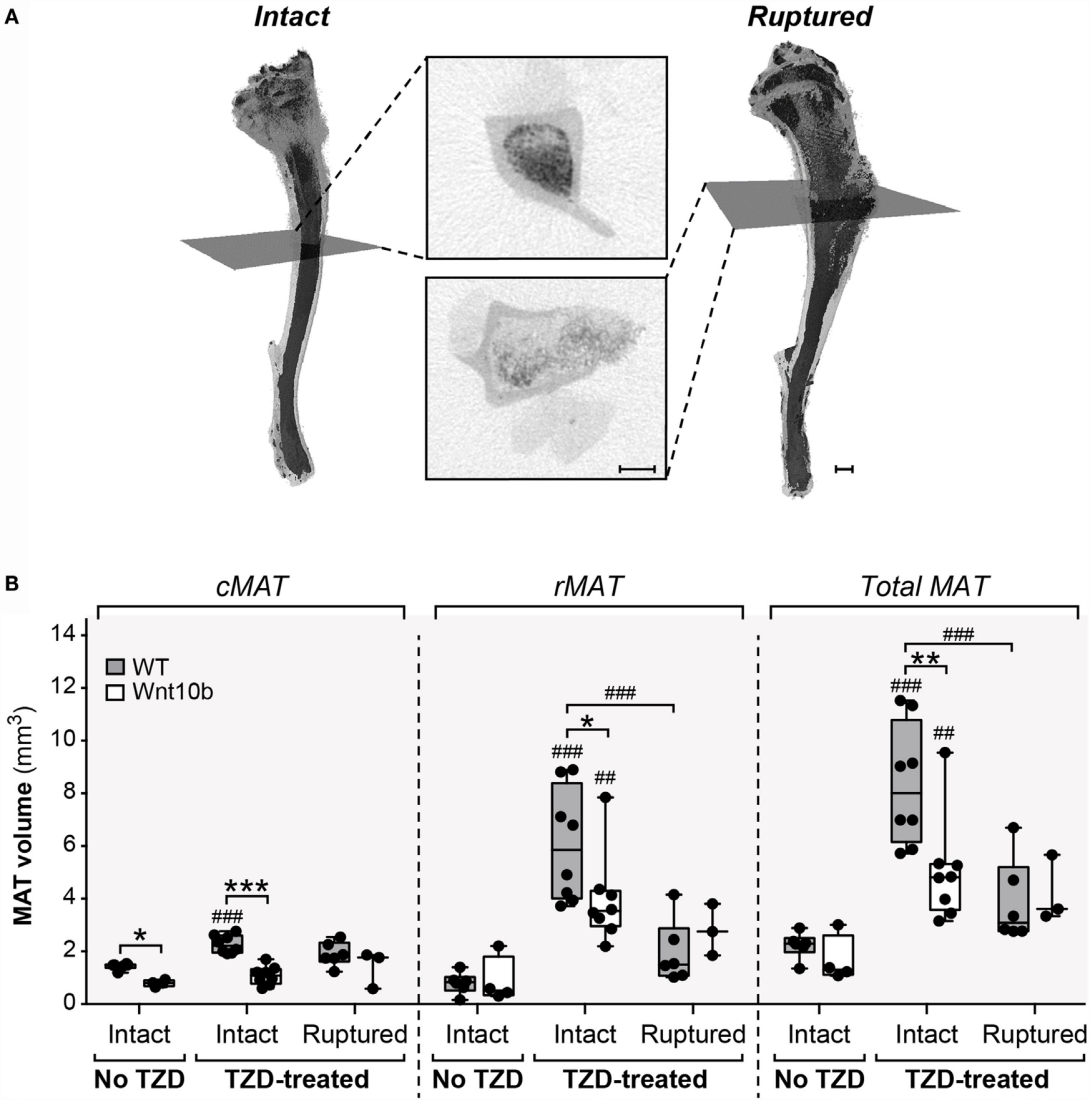
**Cortical bone ruptures significantly decrease detectable rMAT volume**. All male and some female tibiae had ruptured cortical bone near the proximal end, causing a large reduction in signal. **(A)** Representative 3D models of intact (non-ruptured) and ruptured tibiae from 8-week-TZD-treated females, with cross-sectional images showing loss of MAT signal (dark regions within bone marrow cavity). Gray squares across the 3D models indicate the slices from which cross-sectional images are taken. Scale bars = 500 μm. **(B)** Tibiae from female WT or *Ocn*-Wnt10b mice were categorized into bones from mice untreated with TZD, all of which were intact; intact bones from TZD-treated mice; and ruptured bones from TZD-treated mice. The volume of cMAT, rMAT, and total MAT in these bones was determined from μCT scans. Statistically significant differences between untreated (intact), TZD-treated (intact), and TZD-treated (ruptured) samples are indicated by ## (*P* < 0.01) or ### (*P* < 0.001). Within each group, statistically significant differences between WT and *Ocn-*Wnt10b mice are indicated by * (*P* < 0.05), ** (*P* < 0.01), or *** (*P* < 0.001).

The above observations suggest that *Ocn*-Wnt10b males and females partially resist TZD-induced MAT expansion, predominantly as a result of a lower cMAT volume than WT mice. Indeed, rMAT volume did not differ between genotypes in any of the TZD treatment groups. However, when analyzing the μCT scans, we noticed that the decalcified cortical bone had ruptured in tibiae from all male mice and in a subset of females. The ruptures always occurred in proximal tibial diaphysis, as indicated by representative scans of non-ruptured (intact) and ruptured tibiae (Figure 3A). Cross sections show that ruptures allowed escape of the bone marrow, resulting in decreased osmium staining compared to intact tibiae (Figure 3A). This loss of signal suggests that the ruptures might have confounded measurement of MAT volume. To address this, we quantified the volumes of cMAT, rMAT, and total MAT in female tibiae, comparing bones from non-TZD-treated mice (all of which were intact) with intact or ruptured bones from TZD-treated mice. The volume of cMAT did not differ between intact and ruptured bones (Figure 3B), indicating that these ruptures did not affect detection of cMAT in the distal tibia. However, in TZD-treated WT mice, the detectable volume of rMAT and total MAT was significantly lower in ruptured compared to intact bones (Figure 3B). Indeed, in both WT and *Ocn-*Wnt10b mice, TZD-induced expansion of rMAT and total MAT was strongly significant for intact bones, but not when ruptures were present (Figure 3B). This impaired rMAT detection likely explains why TZD-induced rMAT expansion was less marked in male than in female mice (Figure 2B vs. Figure 2E), because all male bones were ruptured. Importantly, for intact tibiae, TZD-induced expansion of rMAT and total MAT was significantly blunted in *Ocn-*Wnt10b compared to WT mice (Figure 3B). This suggests that, in addition to decreased cMAT (Figures 2B,E), *Ocn-*Wnt10b mice are at least mildly resistant to rMAT expansion during TZD treatment.

### Adipocyte Transcript Expression in Tibiae Confirms TZD-Induced MAT Expansion

**Figure 4:**
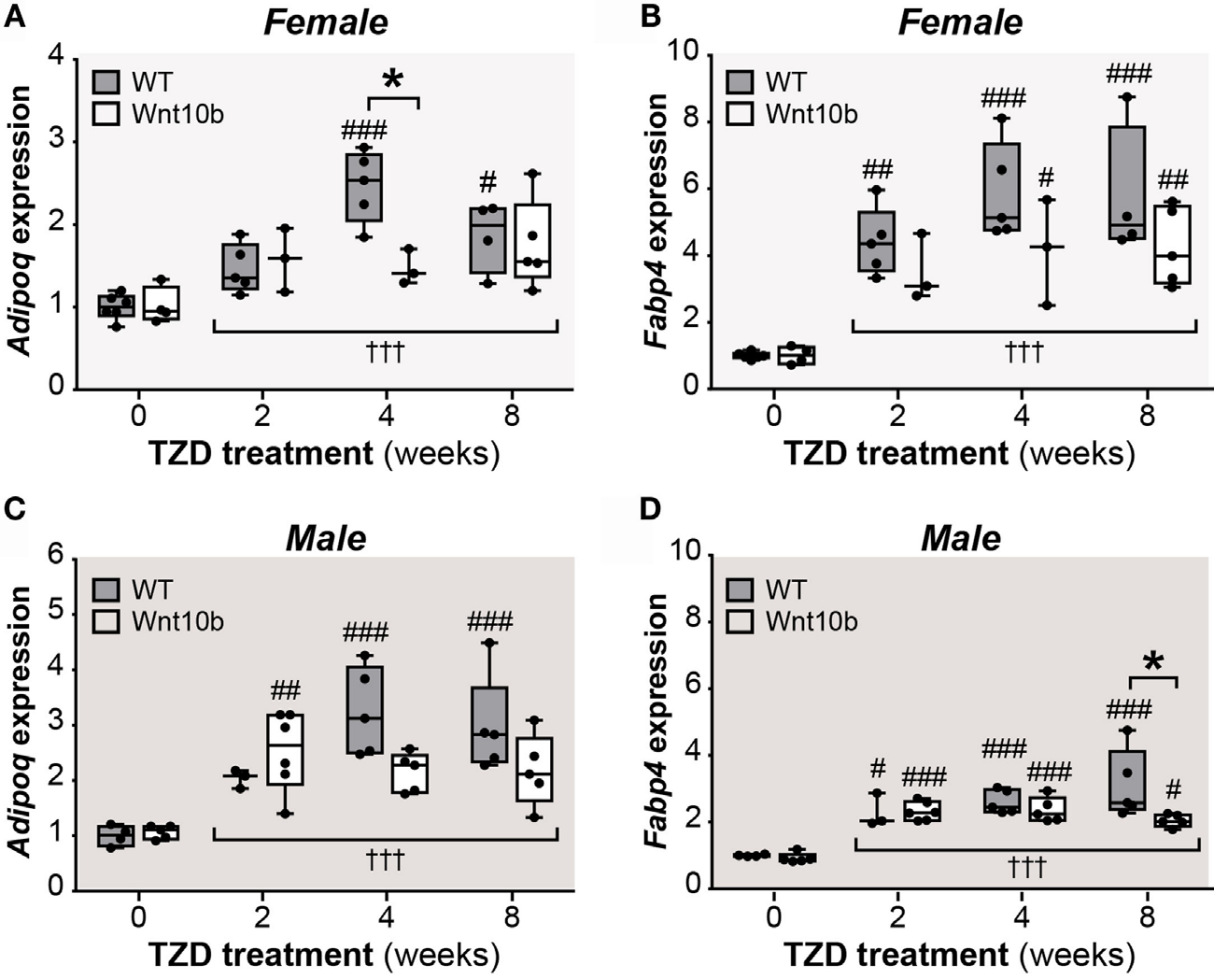
**TZD increases adipocyte marker expression in tibiae, suggesting MAT expansion**. Male and female tibiae were dissected at necropsy, and total RNA was isolated from one tibia of each mouse. Expression of *Adipoq* **(A,C)** and *Fabp4* transcripts **(B,D)** in female and male mice was determined by qPCR and is presented normalized *Ppia* mRNA expression. Statistical significance is presented, as described in Figure 2, for the influence of TZD across all groups, and for differences between TZD-treated and untreated mice (within each genotype) or WT and *Ocn*-Wnt10b mice (within each TZD group).

Given the confounding influence of these bone ruptures, we next pursued other approaches to further assess tibial MAT content. MAT expansion during CR or rosiglitazone treatment has previously been monitored by qPCR analysis of adipocyte marker expression in intact bones, including transcripts for adiponectin (*Adipoq*) and fatty acid-binding protein 4 (*Fabp4*) (3, 26). Thus, we next used qPCR to assess *Adipoq* and *Fabp4* expression in whole tibiae. As shown in Figures 4A–D, across all groups of female or males, expression of *Adipoq* and *Fabp4* was significantly affected by TZD treatment (*P* < 0.0001 for each). Multiple comparisons confirmed that 4- or 8-week TZD significantly increased *Adipoq* in WT males and females (Figures 4A,C) and *Fabp4* in males and females of each genotype (Figures 4B,D). TZD treatment for 2 weeks was also associated with increased *Adipoq* in *Ocn-*Wnt10b males (Figure 4C); increased *Fabp4* in WT females (Figure 4B); and increased *Fabp4* in males of each genotype (Figure 4D). Transcript expression within each TZD treatment group generally did not differ between WT and *Ocn-*Wnt10b mice, with the exception of decreased *Adipoq* in 4-week TZD females (Figure 4A) and decreased *Fabp4* in 8-week TZD males (Figure 4D). However, two-way ANOVA revealed that genotype significantly influenced *Fabp4* in females (*P* = 0.0184) and males (*P* = 0.0339), while there was a significant genotype–TZD interaction that influenced *Adipoq* expression in females (*P* = 0.0193) and males (*P* = 0.0297).

Collectively, these results suggest that TZD increases tibial adipocyte content and that there is some level of resistance to this effect in *Ocn-*Wnt10b mice. Thus, together with the osmium tetroxide analyses of MAT volume (Figures 2 and 3), it seems likely that *Ocn-*Wnt10b mice partially resist TZD-induced MAT expansion, but this is not prevented entirely.

### TZD Induces Hyperadiponectinemia to a Similar Extent in WT and *Ocn*-Wnt10b Mice

**Figure 5:**
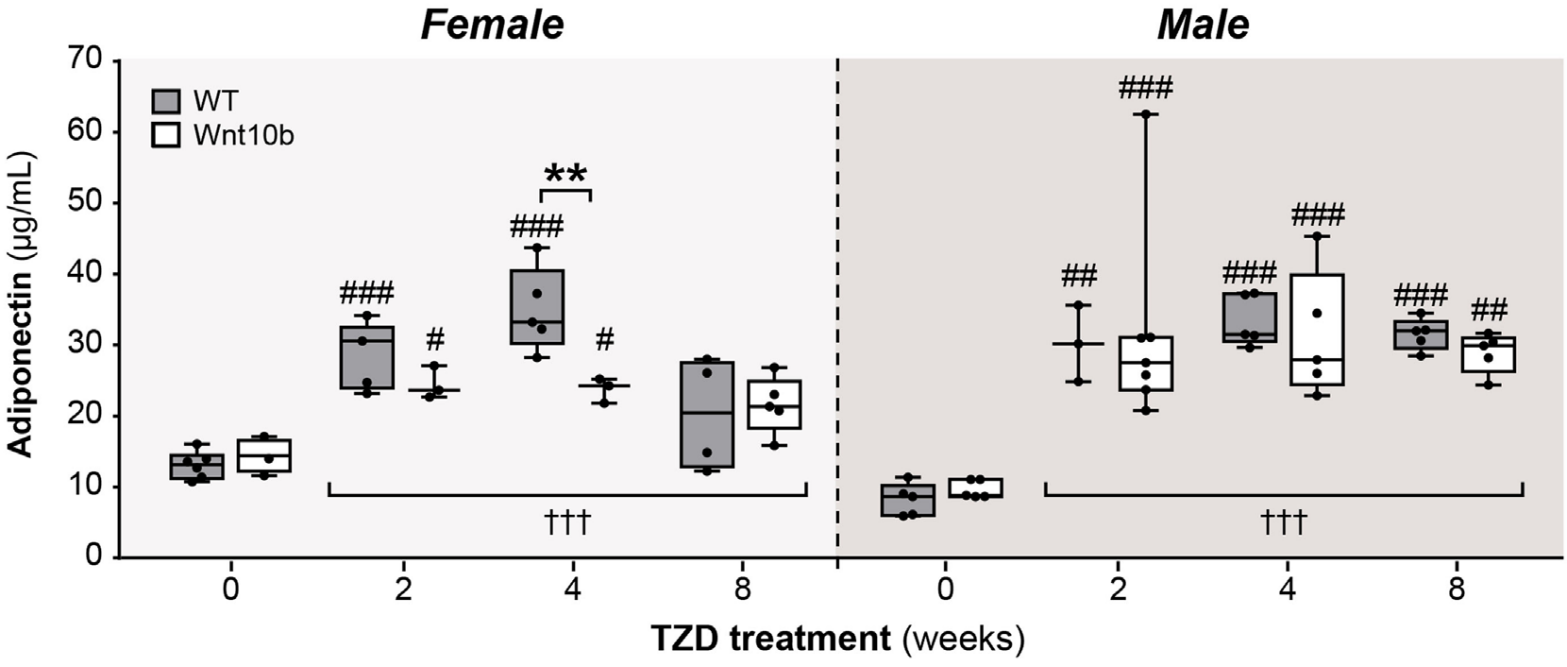
**TZD induces hyperadiponectinemia to a similar extent in WT and *Ocn*-Wnt10b mice**. Serum was isolated from mice at 30 weeks of age and ELISA used to determine concentrations of total adiponectin in female and male mice, as indicated. For each sex, statistical significance for the influence of TZD across all groups, and for differences between TZD-treated and untreated mice (within each genotype) or WT and *Ocn*-Wnt10b mice (within each TZD group), are presented as described for Figure 2.

Given this mild resistance of *Ocn-*Wnt10b mice to MAT expansion, we next investigated if TZD-induced hyperadiponectinemia was altered in these mice. To do so, we used an ELISA to measure serum adiponectin concentrations. Across both genotypes and all TZD groups, treatment with TZD was associated with significantly increased circulating adiponectin (*P* < 0.0001 for males or females) (Figure 5). Genotypic differences were not detected in males; however, in females, there was a significant influence of genotype (*P* = 0.0466) and a significant interaction between genotype and TZD treatment (*P* = 0.0248), indicating that effects of TZD differ between WT and *Ocn-*Wnt10b females. Consistent with this, multiple comparisons showed significantly decreased circulating adiponectin in *Ocn-*Wnt10b compared to WT females following 4 weeks’ TZD (Figure 5). Thus, while TZD-induced hyperadiponectinemia was similar between WT and *Ocn-*Wnt10b males, this effect of TZD was mildly blunted in female *Ocn-*Wnt10b mice.

### TZD Increases Adiponectin Protein Expression in WAT

**Figure 6:**
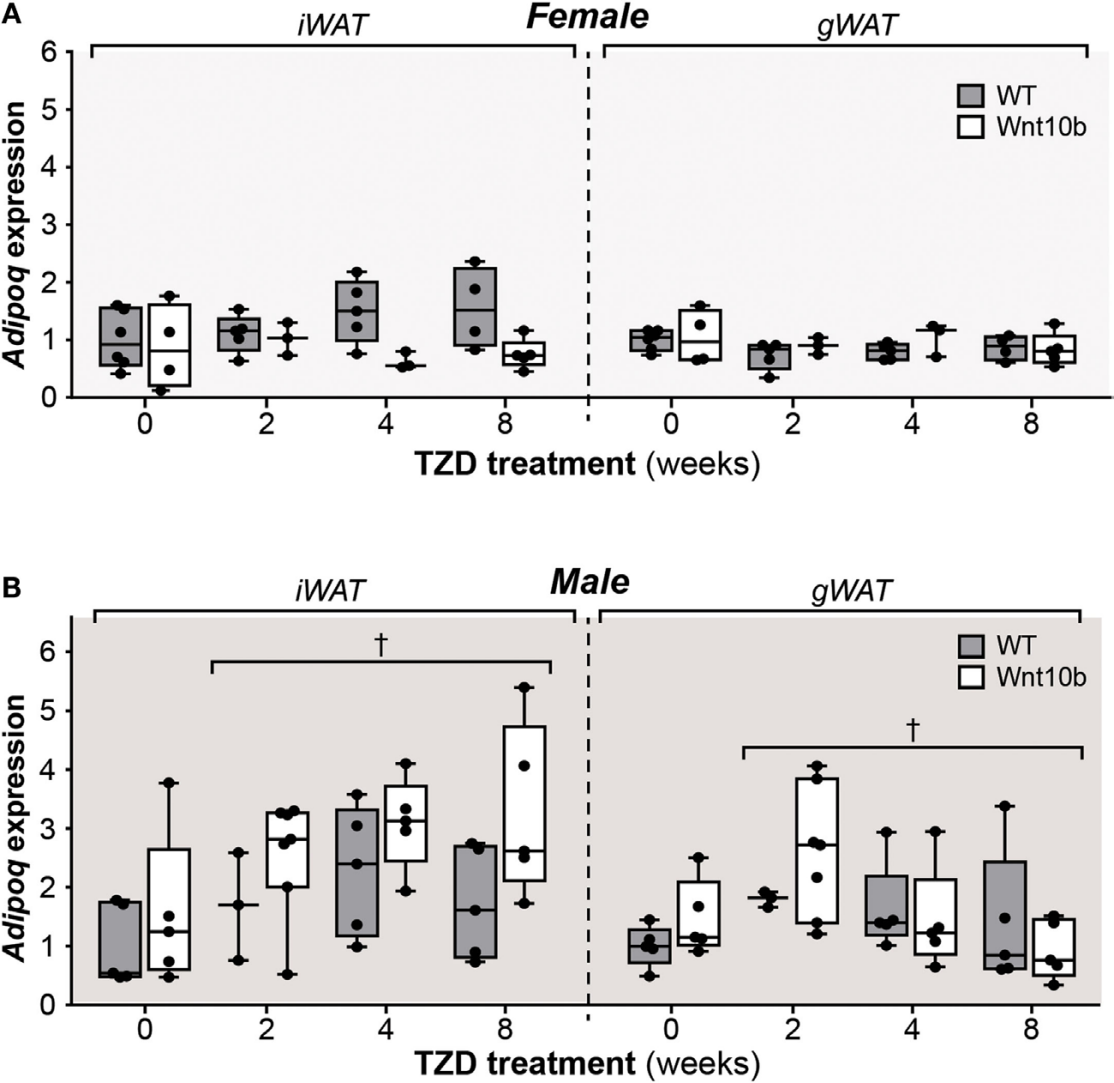
**Rosiglitazone treatment or Wnt10b transgene expression does not alter adiponectin transcript expression in WAT**. At necropsy, iWAT and gWAT were dissected, and total RNA was isolated from portions of each tissue. Expression of *Adipoq* in female **(A)** and male mice **(B)** was determined by qPCR and is presented normalized *Ppia* mRNA expression. Across all groups, statistically significant effects of TZD are presented as described for Figure 2, while significant effects of genotype are described in the text. There were no statistically significant differences between TZD-treated and untreated mice (within each genotype) or between WT and *Ocn*-Wnt10b mice (within each TZD group), as determined by two-way ANOVA.

The above observations demonstrate that, despite having significantly decreased cMAT and being partially resistant to TZD-induced rMAT expansion, *Ocn-*Wnt10b mice undergo hyperadiponectinemia to a similar extent to their WT counterparts. Thus, factors beyond MAT might have a greater influence over TZD-induced hyperadiponectinemia. To address this possibility, we next investigated the effects of TZD and genotype on adiponectin expression in WAT. In WT and *Ocn-*Wnt10b females, TZD did not influence *Adipoq* expression in iWAT or gWAT, either when comparing individual treatment durations or when effects were assessed across all groups (Figure 6A). However, in female iWAT, *Adipoq* transcripts varied significantly by genotype (*P* = 0.0095), with expression diverging as TZD duration increased (Figure 6A). Given that Wnt10b transgene expression is restricted to osteoblasts, this genotypic difference in WAT was unexpected. Across all groups of males, TZD treatment had a minor influence on *Adipoq* transcript levels in both iWAT and gWAT (*P* = 0.0269 and *P* = 0.0461, respectively), although multiple comparisons found no significant effects of TZD between individual treatment groups (Figure 6B). As for females, male iWAT also exhibited genotypic variation (*P* = 0.0096), although in this case *Adipoq* expression tended to be greater in *Ocn-*Wnt10b than in WT mice (Figure 6B).

**Figure 7:**
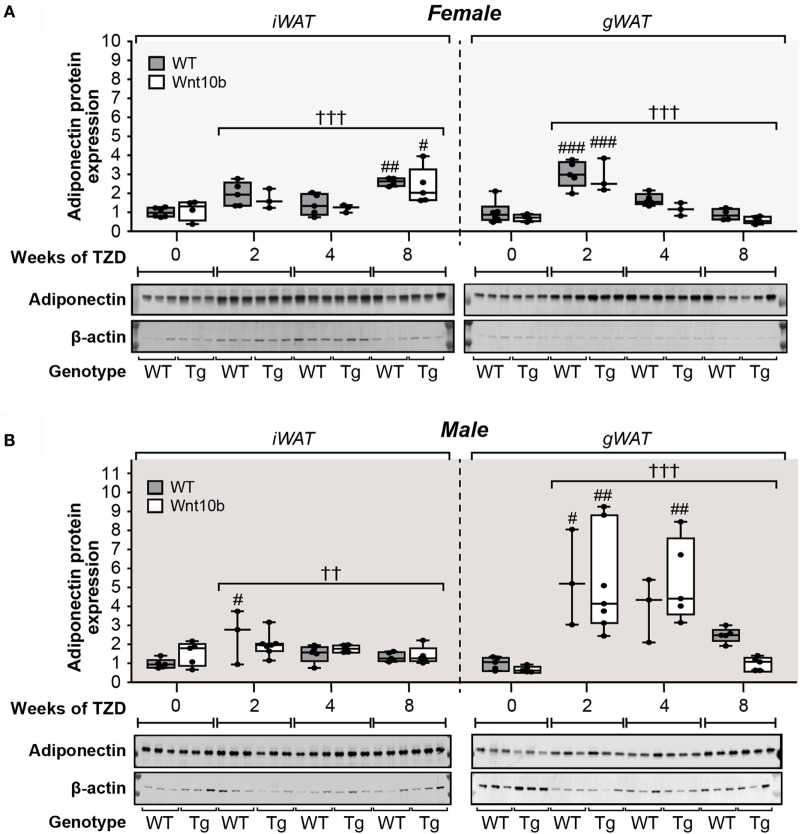
**Rosiglitazone increases adiponectin protein expression in WAT in a depot- and sex-specific manner**. At necropsy, iWAT and gWAT were dissected, and total protein was isolated from portions of each tissue. Lysates were separated by SDS-PAGE and expression of adiponectin protein determined by immunoblotting; β-actin expression was analyzed as a loading control. Immunoblots shown in **(A,B)** are representative of three mice per group. Samples from *Ocn*-Wnt10b mice are labeled “Tg.” Band intensities from these blots and further blots of the remaining samples were quantified using Image Studio Lite software. Adiponectin expression was then normalized to expression of β-actin for **(A)** Males and **(B)** Females, with representative blots shown beneath. For each sex, statistically significant differences between TZD-treated and untreated mice (within each genotype) or between WT and *Ocn*-Wnt10b mice (within each TZD group) are presented as described for Figure 2.

These findings show that WAT *Adipoq* expression is influenced only modestly by TZD, in contrast to the marked TZD-mediated increases in circulating adiponectin (Figure 5). Indeed, our above observations in female mice are consistent with previous reports demonstrating that TZDs can increase circulating adiponectin without increasing *Adipoq* expression in WAT (23, 24). However, other studies have reported a disparity between adiponectin expression at the transcript and protein level (32, 33). Therefore, we next used fluorescence-based immunoblotting to detect and quantify adiponectin protein expression. As shown in Figure 7A, across all groups of female mice, there was a significant influence of TZD on adiponectin protein expression in iWAT and gWAT (*P* < 0.0001 for both), with adiponectin typically increased in WAT of TZD-treated mice compared to untreated controls. Multiple comparisons further confirmed that, for females of each genotype, TZD treatment for 2 or 8 weeks significantly increased adiponectin protein in gWAT or iWAT, respectively (Figure 7A). As for females, across all groups of male mice, TZD had a significant influence on adiponectin protein in iWAT (*P* = 0.004) and gWAT (*P* = 0.0004) (Figure 7B). Based on multiple comparisons, adiponectin was significantly elevated in iWAT and gWAT of WT males after 2 weeks of TZD, and in gWAT of *Ocn*-Wnt10b males after 2 or 4 weeks of TZD (Figure 7B). These results demonstrate that rosiglitazone significantly increases expression of adiponectin protein in WAT of female and male mice. Thus, it seems likely that, with this regimen of rosiglitazone treatment, WAT makes at least a partial contribution to TZD-induced hyperadiponectinemia.

### TZD Induces UCP1 Protein Expression in BAT but Not in WAT or Tibiae

While these studies focused on the relationship between MAT and adiponectin, they also provided the opportunity to assess other properties of MAT; indeed, it remains unclear to what extent MAT’s characteristics overlap with those of WAT and BAT. The key function of BAT is to mediate adaptive thermogenesis *via* uncoupled respiration, which is dependent on expression of uncoupling protein 1 (UCP1). TZDs dose-dependently increase UCP1 protein and *Ucp1* transcripts in BAT and can upregulate *Ucp1* in WAT and whole tibiae (26, 34). Based on the latter, it has been suggested that MAT may have BAT-like characteristics (26). However, others have argued that elevated *Ucp1* expression alone, without assessment of UCP1 protein, is insufficient evidence for a tissue’s thermogenic capacity (35). Indeed, the relative protein expression of UCP1 between MAT and BAT remains to be firmly established. Thus, to further study the BAT-like properties of MAT, we next analyzed UCP1 expression in BAT, WAT, and tibiae, both at the transcript and protein level.

**Figure 8:**
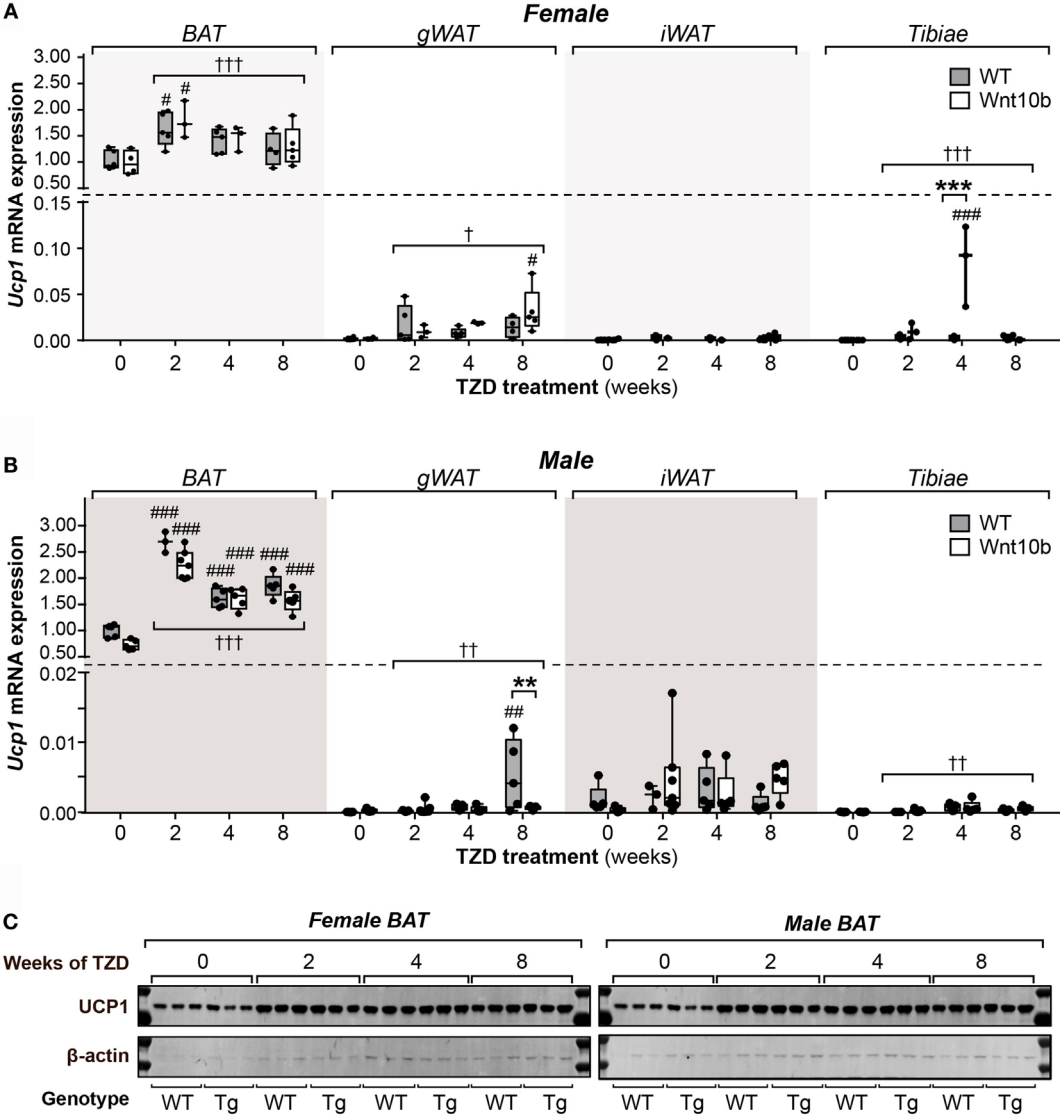
**Rosiglitazone increases UCP1 mRNA and protein in BAT not in WAT or tibiae**. Total RNA and protein were isolated from interscapular BAT, gWAT, iWAT, and whole tibiae. **(A,B)** *Ucp1* transcript expression in each tissue of female **(A)** and male mice **(B)** was quantified by qPCR and normalized to expression of *Ppia* mRNA. In each tissue, statistically significant differences between TZD-treated and untreated mice (within each genotype) or between WT and *Ocn*-Wnt10b mice (within each TZD group) were determined by two-way ANOVA and are presented as described for Figure 2. **(C)** Protein lysates from BAT were separated by SDS-PAGE and expression of UCP1 protein determined by immunoblotting; β-actin expression was analyzed as a loading control. Immunoblots are representative of three mice per group. Samples from *Ocn*-Wnt10b mice are labeled “Tg.” UCP1 protein could not be detected in gWAT, iWAT, or tibiae (data not shown).

As shown in Figures 8A,B, across all groups of female or male mice, TZD strongly influenced *Ucp1* expression in BAT (females, *P* = 0.0002; males, *P* < 0.0001). Multiple comparisons further confirmed that, relative to untreated controls, each duration of TZD significantly increased BAT *Ucp1* in males of each genotype (Figure 8B), while this also occurred for females with 2 weeks’ TZD treatment (Figure 8A). Across-group effects of TZD were also detected in female gWAT (*P* = 0.014), female tibiae (*P* < 0.0001), male gWAT (*P* = 0.0051), and male tibiae (*P* = 0.0017), wherein TZD-treated groups typically had greater *Ucp1* expression than untreated controls (Figures 8A,B). Analysis of genotypic differences *via* multiple comparisons confirmed that, relative to untreated mice, *Ucp1* was significantly elevated in *Ocn-*Wnt10b female tibiae or gWAT after 4 or 8 weeks’ TZD, respectively, and in WT male gWAT, after 8 weeks’ TZD (Figures 8A,B). Each of these groups also had significantly greater *Ucp1* expression than their untreated WT or *Ocn*-Wnt10b counterparts, respectively; however, such genotypic differences were uncommon, and genotype generally did not influence *Ucp1* expression in any of the tissues analyzed.

These data demonstrate that rosiglitazone increases *Ucp1* expression in BAT, gWAT, and tibiae, consistent with results in previous studies (26, 34). However, relative to expression in BAT, *Ucp1* expression in untreated mice was over 500-fold lower in gWAT, 1,500-fold lower in iWAT, and 10,000-fold lower in tibiae (Figures 8A,B). Thus, even with TZD treatment, *Ucp1* expression in these tissues is negligible, raising questions over its functional relevance. To further address this, we next analyzed UCP1 expression at the protein level. As shown in Figure 8C, in females and males of either genotype, TZD robustly increased UCP1 expression in BAT; however, in gWAT, iWAT, or whole tibiae, we could not detect UCP1 protein expression (data not shown). Together, these observations suggest that MAT is unlikely to possess the thermogenic properties of BAT.

## Discussion

Herein, we pursued studies in *Ocn*-Wnt10b mice to investigate the hypothesis that MAT expansion contributes to TZD-induced hyperadiponectinemia. We found that, regardless of TZD treatment, *Ocn-*Wnt10b mice have significantly less cMAT than WT mice and likely resist TZD-induced rMAT accumulation. Despite this loss of cMAT and mild resistance to rMAT expansion, circulating adiponectin is generally similar between WT and *Ocn-*Wnt10b mice. Moreover, we found that TZD significantly increases adiponectin protein expression in WAT. Together, these findings suggest that WAT, rather than MAT, is the key mediator of TZD-induced hyperadiponectinemia, at least under these conditions of TZD treatment. However, as discussed in Section “Contribution of MAT and WAT to TZD-Induced Hyperadiponectinemia,” further consideration of these findings suggests that MAT makes at least some contribution to TZD-induced hyperadiponectinemia. As a secondary aim, we also addressed the hypothesis that MAT has BAT-like properties. We found that, in contrast to BAT, UCP1 protein is undetectable in tibiae, suggesting that MAT is unlikely to have the thermogenic capacity of BAT.

As further discussed below, these findings shed new light on the function of MAT and have implications worth considering for future MAT research.

### Contribution of MAT and WAT to TZD-Induced Hyperadiponectinemia

We previously identified MAT as a source of increased circulating adiponectin during CR, a conclusion based, in part, on the finding that *Ocn-*Wnt10b mice resist both CR-associated MAT expansion and hyperadiponectinemia (2, 3).

Herein, we find that, regardless of TZD treatment, cMAT volume is significantly lower in *Ocn-*Wnt10b than in WT mice. Unfortunately, tibial ruptures confounded measurement of rMAT volume; however, analysis of intact tibiae, without ruptured samples, suggests strongly that *Ocn*-Wnt10b mice also partially resist rMAT accumulation in response to TZD treatment. Despite this loss of cMAT and mild resistance to rMAT expansion, circulating adiponectin is generally similar between WT and *Ocn*-Wnt10b mice. Superficially, this suggests that MAT expansion *per se* does not contribute to hyperadiponectinemia under these TZD treatment conditions. However, this conclusion is less certain when several other key factors are taken into account.

One such factor is the likely contribution of WAT. Unlike during CR, adiponectin protein expression in WAT is increased under these conditions of TZD treatment, which may override any effects of suppressed MAT expansion. These increases are more pronounced for adiponectin protein than for *Adipoq* transcripts, with TZD affecting the *Adipoq* transcripts only in male WAT (Figure 6). These findings echo previous reports of sex-specific differences in WAT adiponectin expression, and of a disconnect between adiponectin expression at the transcript and protein level (32, 33). Notably, these effects on WAT are inconsistent with the finding that TZDs can increase circulating adiponectin without increasing adiponectin expression in WAT (23, 24). This disparity is likely a result of the higher dose of rosiglitazone used in our study. Indeed, WAT adiponectin expression is unaltered by lower TZD doses (24, 36) but significantly increased at higher rosiglitazone concentrations (18). Thus, as discussed further below (“Implications of TZD Dose on the Capacity of *Ocn*-Wnt10b Mice to Resist MAT Expansion and Hyperadiponectinemia”), a lower dose of rosiglitazone would likely be required if we were to further investigate the contribution of MAT to TZD-associated hyperadiponectinemia.

Considering these findings, it seems likely that WAT makes at least some contribution to hyperadiponectinemia under these conditions of rosiglitazone treatment. However, the effect of TZD on WAT adiponectin expression (Figures 6 and 7) is far less consistent and pronounced than that which occurs for circulating adiponectin concentrations (Figure 5). In contrast, these changes in circulating adiponectin are closely reflected by the increases in tibial adiponectin expression (Figure 4). For example, when comparing *Ocn-*Wnt10b and WT mice, circulating adiponectin is lower only in the 4-week TZD females (Figure 5); hence, it is striking that this is also the only group in which tibial adiponectin expression is lower in *Ocn-*Wnt10b mice (Figure 4A). Together, these observations show that circulating adiponectin is closely associated with tibial adiponectin expression and less tightly associated with gross changes in rMAT and cMAT volume. This underscores the importance of analyzing MAT through multiple approaches, including transcriptional markers, rather than focusing solely on MAT volume *via* osmium tetroxide staining. Perhaps, more importantly, this close association suggests that adiponectin production from MAT contributes, at least in part, to TZD-induced hyperadiponectinemia.

### Implications of TZD Dose on the Capacity of *Ocn*-Wnt10b Mice to Resist MAT Expansion and Hyperadiponectinemia

Previous studies have used a wide range of doses of rosiglitazone to investigate its impact on adiponectin or bone marrow adiposity. For example, Nawrocki et al. treated mice with 10 mg/kg/day to examine the effects on circulating adiponectin and glucose homeostasis (20), while 20 mg/kg/day was used in two more recent studies investigating effects of rosiglitazone on bone marrow adiposity (26, 27). A much lower dose of 3 mg/kg/day was also found to cause MAT expansion in mice, but whether this promotes hyperadiponectinemia was not reported (28, 37). Thus, in the present study, we used 15 mg/kg/day to ensure robust effects on MAT and circulating adiponectin. As in the above studies (26–28), we did so by administering rosiglitazone in the diet, based on measurements of body mass and estimated daily food intake. However, because of variation in body masses and daily food consumption, some mice may have exceeded the target dose of 15 mg/kg/day. As discussed above, this relatively high dose may explain the increased adiponectin expression in WAT, thereby complicating interpretation of any contribution from MAT. Such high doses may also have overwhelmed the ability of *Ocn*-Wnt10b mice to more robustly resist MAT expansion. Thus, a lower rosiglitazone dose may limit these confounding effects and thereby be more suitable for addressing our hypothesis in *Ocn-*Wnt10b mice.

### Limitations of *Ocn-*Wnt10b Mice as a Model Resistant to MAT Expansion

One reason that we have not pursued such follow-up studies is that *Ocn*-Wnt10b mice may be too limited a model in which to robustly address MAT’s endocrine functions. For example, while CR- or TZD-induced MAT expansion is blunted in these mice, such expansion is still marked in comparison to untreated *Ocn*-Wnt10b controls (Figures 2 and 3) (3). Moreover, distal tibiae of *Ocn-*Wnt10b mice remain laden with MAT (Figures 2A,D) and, while cMAT volume is lower in *Ocn*-Wnt10b mice, this likely reflects restrictions imposed by decreased bone marrow volume (3), rather than resulting from a direct inhibition of adipogenesis. Thus, as we have recently argued elsewhere (7), future research of MAT would benefit enormously from development of new mouse models that more robustly resist MAT formation, developmentally and/or in response to CR, TZDs, or other stimuli that promote MAT expansion.

### Confounding Effects of Bone Ruptures on Osmium Tetroxide-Based rMAT Analysis

The present study reveals that cortical bone ruptures can confound osmium-based MAT detection. This issue, which has not been reported previously, provides further support for taking a multifaceted approach to MAT analysis. While TZDs can cause bone loss and fractures (38), it is unlikely that the ruptures occurred *in vivo* over the course of rosiglitazone treatment: such an injury would have dramatically impacted mobility and behavior of the mice, which was not apparent during daily inspections; and the ruptures also occurred in tibiae of males untreated with rosiglitazone, demonstrating that they are not a consequence of TZD treatment. Instead, it is likely that ruptures occurred *ex vivo* as a result of methodological issues. In particular, after fixing tibiae in formalin post-necropsy there was a very long time span, ~30 months, before most of these bones were decalcified and analyzed by osmium tetroxide staining. This holdup largely resulted from delays associated with one of our lead authors moving to a new institution, during which time the fixed tibiae were stored in PBS at 4°C and shipped by air from the USA to the UK. Although these storage conditions would not be expected to promote cortical ruptures, it is notable that no ruptures occurred in a subset of female tibiae that were stored in PBS for a shorter duration (~12 months), without air transport, before decalcification and osmium analysis. Moreover, at both the University of Michigan and the University of Edinburgh, we have analyzed hundreds of osmium-stained mouse bones from other studies, none of which has undergone such long-term storage or air transport, and among which no ruptures have ever been detected. Thus, one possibility is that exposure to low air pressure during air transport can cause a pressure differential that promotes bone ruptures. It remains possible that other factors also contributed to this phenomenon; however, our findings strongly suggest that fixed bones should not undergo long-term storage or exposure to low atmospheric pressure if MAT volume is to be determined accurately using osmium tetroxide staining.

### BAT-Like Thermogenic Function Is Unlikely in MAT

There is extensive interest in “beige” or “brite” (brown-in-white) adipocytes, which develop in WAT in response to diverse external stimuli, including cold exposure or TZD treatment, and which share some properties of brown adipocytes (39–41). This so-called “browning” of WAT can increase energy consumption and may thereby contribute to TZD-associated improvement in metabolic health. Therefore, whether MAT can also undergo browning or has BAT-like characteristics has been a subject of some interest. Krings et al. found that, in mice treated for 4 weeks with rosiglitazone (20 mg/kg/day), transcript expression of brown adipocyte markers is increased in tibiae, supporting the possibility that MAT has BAT-like characteristics (26). This is consistent with our finding that rosiglitazone influences tibial *Ucp1* mRNA expression (Figure 8). However, Krings et al. further highlighted that, compared to BAT, *Ucp1* expression is 16,000-fold lower in tibiae, which agrees closely with our data showing a 10,000- to 25,000-fold decrease in tibiae compared to BAT. In addition, even with rosiglitazone treatment, we find that *Ucp1* transcripts remain 400- to 7,000-fold lower in tibiae than in BAT (Figures 8A,B). Crucially, at this level of *Ucp1* expression, we could not detect UCP1 protein, whereas rosiglitazone-induced increases in UCP1 protein in BAT are readily detectable (Figure 8C).

UCP1 is the hallmark of brown adipocytes and the key mediator of adaptive thermogenesis, and therefore the above findings strongly suggest that bone marrow adipocytes are unlikely to possess BAT-like thermogenic properties. One limitation of our study, and that of Krings et al., is that we analyzed whole bones rather than isolated marrow adipocytes. However, both brown and beige adipocytes are further defined by their high mitochondrial content and the multilocular morphology of their lipid droplets (41), neither of which is observed in bone marrow adipocytes (3, 4, 12, 42). This raises further doubts about the notion that MAT has BAT-like characteristics, an issue that we recently discussed in greater depth elsewhere (5). Nevertheless, it remains possible that, under a different TZD treatment regimen or in response to other stimuli, MAT is capable of undergoing browning. Indeed, while we were unable to detect UCP1 protein in WAT or whole tibiae, previous studies have detected UCP1 in WAT following TZD treatment (41). Moreover, effects of TZDs on *Ucp1* expression in BAT are dose-dependent (34). This supports the possibility that TZD-mediated browning of WAT and MAT requires an optimal regimen of TZD treatment, in which case the conditions in our study may simply have been unsuitable for inducing a browning response. Thus, there is ample scope for future studies to further investigate whether MAT has BAT-like properties, both at the molecular and functional levels.

### Other Strengths and Limitations of This Study

This study is the first to address the novel hypothesis that MAT contributes to hyperadiponectinemia in conditions beyond CR. The experimental design is strengthened by including both male and female mice, as well as by analysis of several durations of TZD treatment. The discovery of the confounding effects of cortical ruptures is partly a strength, in that it highlights an important technical consideration for future MAT research; however, presently this was a limitation because it prevented thorough measurement of rMAT volume. As discussed above, another limitation is that the dose of rosiglitazone may have been too high, and may have varied slightly between mice because of differences in body mass and daily food intake. Administration of lower doses by daily injection or oral gavage could be one approach to overcome this issue. Finally, a key limitation of this study is that our findings are based on only a single cohort of mice. Ideally, these experiments would be repeated in a second cohort, with particular care taken to avoid cortical ruptures; this would allow more robust determination of rMAT volume in *Ocn-*Wnt10b mice. However, as noted above (“Limitations of *Ocn*-Wnt10b Mice as a Model Resistant to MAT Expansion”), *Ocn-*Wnt10b mice may not be sufficiently robust as a model of impaired MAT formation. For example, adipocyte marker expression in tibiae is generally similar between WT and *Ocn*-Wnt10b mice (Figure 4). This limitation of the *Ocn-*Wnt10b model undermines the rationale for repeating the present studies in a second cohort. Instead, we feel strongly that it would be more productive and scientifically beneficial to focus efforts on developing new mouse models that more robustly resist MAT expansion. Such models could then be used to better address the present hypothesis, as well as other aspects of MAT function.

Beyond these particular issues, one broader limitation is that our experiments were based only in mice; it remains unclear if the relationship between TZD-induced MAT expansion and hyperadiponectinemia also exists in humans. It is well established that TZDs increase circulating adiponectin in humans (16), but their effects on MAT are less clear. Indeed, one study finds that rosiglitazone *decreases* bone marrow adiposity in the lumbar vertebrae (43), whereas a more recent report finds that pioglitazone, another TZD, increases femoral and lumbar vertebral bone marrow adiposity (44). Unfortunately, neither of these studies assessed circulating adiponectin, and there remain very few clinical studies assessing the effect of TZDs on MAT. Thus, there is a strong rationale for future research to establish how TZDs affect MAT accumulation in humans and whether this is associated with changes in circulating adiponectin.

## Conclusion

There are four main conclusions from our study. First, under these TZD treatment conditions, it is likely that both WAT and MAT make some contribution to TZD-induced hyperadiponectinemia. Second, UCP1 expression in MAT is negligible, and therefore MAT is unlikely to have the thermogenic capacity of BAT. Third, cortical bone ruptures confound measurement of MAT volume by osmium tetroxide staining, and therefore care must be taken when processing bones for such analysis. Moreover, osmium-based assessment of MAT volume can differ from measurement of MAT content based on expression of bone marrow adipocyte transcripts; hence, multiple approaches should be used to assess MAT content, including analysis of molecular markers, rather than relying solely on osmium tetroxide staining. Finally, while *Ocn-*Wnt10b mice have been useful as a model resistant to MAT formation, development of more robust loss-of-MAT models would be of great benefit to future MAT research.

## Author Contributions

RS and WC designed all figures and wrote the manuscript, with ES, HM, and OM providing additional critical revisions. WC, BL, ES, VK, and OM contributed to the conception and design of the experiments. BL oversaw mouse breeding and colony management. RS, BL, BZ, ES, SP, BS, HM, AB, RW, OM, and WC contributed to data acquisition, analysis, and/or interpretation. All authors gave final approval for publication of the manuscript and agree to be accountable for all aspects of the work presented herein.

## Conflict of Interest Statement

RS, BL, BZ, ES, SP, BS, HM, AB, and RW have nothing to disclose. WC held a postdoctoral fellowship funded by Eli Lilly and Company. VK is employed by Eli Lilly and Company. OM has received research funding from Eli Lilly and Company.
